# MiR‐101 promotes pain hypersensitivity in rats with chronic constriction injury *via* the MKP‐1 mediated MAPK pathway

**DOI:** 10.1111/jcmm.15532

**Published:** 2020-07-13

**Authors:** Shuang Qiu, Benjuan Liu, Yanshuai Mo, Xueqin Wang, Lina Zhong, Xiao Han, Fuli Mi

**Affiliations:** ^1^ Department of Anesthesiology Linyi People's Hospital Linyi China

**Keywords:** chronic constriction injury, MicroRNA‐101, mitogen‐activated protein kinase phosphatase 1, pain hypersensitivity, spinal cord microglial cell

## Abstract

This study was performed to characterize the effect of microRNA‐101 (miR‐101) on the pain hypersensitivity in CCI rat models with the involvement of mitogen‐activated protein kinase phosphatase 1 (MKP‐1) in spinal cord microglial cells. The mechanical withdrawal threshold (MWT) and thermal withdrawal latency (TWL) in the developed CCI models were determined to assess the hypersensitivity of rats to mechanical stimulation and thermal pain. To assess inflammation, the levels of interleukin (IL)‐1β, IL‐6 and tumour necrosis factor‐α (TNF‐α) in the spinal dorsal horns of CCI rats and lipopolysaccharide (LPS)‐activated microglial cells were examined. miR‐101 and MKP‐1 gain‐ and loss‐of‐function experiments were conducted in in vivo and in vitro settings to examine the roles of miR‐101 and MKP‐1 in CCI hypersensitivity and inflammation. The results showed that miR‐101 was highly expressed in the spinal dorsal horn and microglial cells of CCI rat models. Furthermore, overexpression of miR‐101 promoted the pain hypersensitivity in CCI rat models by reducing MWT and TWL. The overexpression of miR‐101 also promoted inflammation in LPS‐exposed microglial cells, as indicated by increased levels of IL‐1β, IL‐6 and TNF‐α. MiR‐101 was shown to target MKP‐1, inhibiting its expression. Moreover, miR‐101 promoted pain hypersensitivity in CCI rat models by inhibiting MKP‐1 expression and activating the mitogen‐activated protein kinase (MAPK) signalling pathway. Taken together, miR‐101 could potentially promote hypersensitivity and inflammatory response of microglial cells and aggravate neuropathic pain in CCI rat models by inhibiting MKP‐1 in the MAPK signalling pathway.

## 1 INTRODUCTION

Chronic pain pre‐disposes approximately 20% of the population across the world to physical and emotional incapacity and is regarded as one of the leading causes of disability.^1^ Notably, approximately, 20% of the cases of chronic pain comprise neuropathic pain arising from impairment of the nervous system, which may be attributed to direct damage to the spinal cord, the brain or the peripheral nerves, or dysfunction due to degenerative or chronic inflammation.^2^ In particular, the activation of spinal cord microglial cells is responsible for persistent neuropathic pain.^3^ The treatment strategies for nerve injury and subsequent neuropathic pain are usually aimed at reducing inflammation, a primary cause underlying neuropathic pain.^4^ However, neuropathic pain is a frequently occurred condition, which often has poor treatment outcome due to resistance to therapies.^5^ The genesis and development of neuropathic pain are still poorly understood, and thus current therapies only focused on symptomatic management instead of targeting the underlying aetiology.^6^ Therefore, the search on more effective and safer treatment options for neuropathic pain warrants investigations into its relevant molecular mechanisms.

MicroRNAs (miRNAs) are a group of small non‐coding RNAs and known to mediate gene expression through either translational inhibition or mRNA degradation.^7^ Interestingly, multiple miRNAs have been reported as molecular players in the generation and development of chronic neuropathic pain^8^ owing to their roles as key modulators of gene expression and neuronal network plasticity within the nervous system.^9^ The aberrant expression of miRNAs has been observed in the spinal cord dorsal horn in rat models of chronic constriction injury (CCI) and plays important roles in the induction and persistence of neuropathic pain.^10^, ^11^ Interestingly, a previous study has indicated that miR‐101a could induce the differentiation of bone marrow cells into microglial cells, whose activation has been associated with neuropathic pain.^12^ Moreover, miR‐101 has been demonstrated to attenuate neuropathic pain in CCI rat models.^13^ The interaction of miRNAs and mRNAs has been characterized in inflammatory pain in the rat spinal cord and is also implicated in pain regulating pathways.^14^ miR‐101 has been reported to negatively regulate mitogen‐activated protein kinase phosphatase 1 (MKP‐1) expression.^15^ MKP‐1 is considered a key factor in the mitogen‐activated protein kinase (MAPK) signalling pathway.^16^ As a mediator of phosphorylated p38, MKP‐1 exerts regulatory effects on pro‐inflammatory factor in the spinal cord following peripheral nerve injury, thereby regulating chronic mechanical hypersensitivity.^17^ Inactivation of the MAPK signalling pathway has been documented to attenuate neuropathic pain in CCI rats.^18^ Therefore, we hypothesized that miR‐101 might participate in the induction of pain hypersensitivity following CCI through MKP‐1‐mediated MAPK signalling pathway.

## 2 MATERIALS AND METHODS

### 2.1 Ethics statement

Animal experiments were performed with the approval of the Ethics Committee of Linyi People's Hospital and in accordance with *the Guide for the Care and Use of Laboratory animals* published by the US National Institutes of Health. All efforts were made to minimize the suffering of the animals included in the study.

### 2.2 Rat CCI model establishment

Adult female Sprague Dawley (SD) rats (weighing 180‐210 g) were purchased from Shanghai, Lab. Animal Research Center. All rats were housed in cages with a constant temperature of 25°C.

The CCI rat model was developed according to previously described methods.^19^ Briefly, the rats were anaesthetized by intraperitoneal injections of 40 mg/kg pentobarbital sodium. The sciatic nerves on both sides of the rats were exposed and isolated from the surrounding tissues. The posterior medial sciatic nerve of the left femur was exposed, set free for 5‐6 mm before bifurcating and ligated 4 times within l mm. After the surgery, the incision was sutured layer by layer. Rats in the sham group were subjected to exposure and isolation of the sciatic nerve without ligation. There were 18 rats in the sham group and CCI group (without infection with lentivirus), respectively. The rats developed with CCI were then infected with lentiviruses expressing miR‐101, miR‐101‐inhibitor, MKP‐1, negative control (NC), namely, LV‐miR‐101, LV‐miR‐101‐inhibitor, LV‐MKP‐1 and LV‐NC, with 9 rats in each group. The remaining rats developed with CCI were treated with MKP‐1 inhibitor, RO318220 or infected with LV‐miR‐101 and LV‐MKP‐1 or LV‐NC in combination, with 9 rats in each group. Three rats were randomly selected and euthanized at each time when the spinal cord tissue was harvested, and six randomly selected rats were used for behavioural tests. Next, L4‐L6 spinal cord segment was harvested on days 0, 3, 7, 14 and 21 after the CCI induction surgery.

### 2.3 Intrathecal catheterization and injection

The rats were intraperitoneally anaesthetized with 40 mg/kg pentobarbital sodium. Thereafter, the occipital muscles were separated, and the PE‐10 polyethylene catheter was placed in the cisterna magna of the cerebellum. Next, 80 000 units of penicillin sodium were injected into the right upper extremity to prevent infection. On the next day, after the rats were awake, 20 μL of 2% lidocaine was injected through a microcatheter, and both lower extremities were paralysed within 30 seconds. The rats that recovered within 30 minutes were considered to have a successful catheterization. After catheterization for 2 days, the rats with movement and sensory disturbances such as monoplegia, paraplegia and hemiplegia were excluded. Three days prior to CCI surgery, 10 μL of the corresponding recombinant lentivirus was injected through the intrathecal catheter using a micro‐injector.

### 2.4 Behavioural test

Von Frey's method was used to measure the mechanical withdrawal threshold (MWT) to evaluate the mechanical abnormal pain. The rats were kept in a clear plastic box with a metal mesh bottom. Pressure was applied to the rat hind paw using Calibrated Electronic von Frey filaments (Electronic von Frey 2393; IITC) and the time for paw withdrawal was recorded. Thermal withdrawal latency (TWL) was measured using a 336 analgesic model (IITC Life Science Instruments) to assess thermal hyperalgesia. The rats were kept in a plexiglass box, and in order to avoid tissue damage caused by long‐term thermal stimulation, the heat intensity was set to 10 seconds, and the cut‐off time was 20 seconds (the power was stopped automatically 30 seconds later). The duration between stimulation and withdrawal was recorded. Each test was repeated 5 times at an interval of 5 minutes for each claw, with the mean value obtained. At the end of the behavioural test, the rats were euthanized, and bilateral L4‐L6 spinal segments were harvested in a chronological order.

### 2.5 Lentiviral vector construction and transfection

Full‐length miR‐101 and its inhibitor, MKP‐1 oligonucleotide or NC oligonucleotide were subcloned into GV280 lentiviral vectors (GeneChem) to construct LV‐miR‐101, LV‐miR‐101‐inhibitor, LV‐MKP‐1 and LV‐NC vectors. The lentivirus was collected and purified using the ultracentrifugation method. Recombinant lentivirus with 8 μg/mL polybrene (Sigma) was injected into the rats *via* an intrathecal catheter using a microinjection syringe.

Spinal cord microglial cells (1 × 10^6^) were treated with 50 μg of miR‐101 plasmid or inhibitor or NC in 100 μL Lipofectamine™ 2000 transfection reagent (11668019, Invitrogen) in accordance with kit instructions. The mixture was incubated at 37°C for 6 hours, and the old medium was removed. The cells were further incubated for 24‐48 hours with complete medium. RNA was extracted and transfection efficiency was verified for subsequent experiments.

### 2.6 Cell separation and culture

The isolation of rat spinal cord microglial cells was performed as described in previous study.^20^ SD rats in the sham and CCI groups were euthanized and the spinal cord tissues at enlarged lumbar region were harvested. The lumbar spinal cord was immersed in 4 mL ice‐cold Hank's solution containing 15 mmol/L HEPES (Gibco) and 0.5% glucose (Sigma‐Aldrich) and ground. The suspension was then filtered through a sterile cell strainer (70 μL; BD Biosciences). Cells were collected and centrifuged at 400 *g* for 10 minutes. The cell supernatant was harvested. The 15 mL centrifuge tube was added with 3 mL 75% Percoll, 3 mL 50% Percol and 3 mL 5% Percol and 2 mL phosphate‐buffered saline (PBS) in successive. Next, the cell supernatant was loaded into this centrifuge tube for 20‐minute centrifugation at 1000 *g*. The cells at the 50/75% interface (the mixture of microglial cells and other types of cells) were collected. The purity of microglial cells was identified using immunofluorescence. The harvested cells were rinsed in ice‐cold PBS and then resuspended in PBS supplemented with 1% bovine serum albumin (BSA) for further analysis. HEK‐293T cells used in this study were purchased from American Type Culture Collection (ATCC; https://www.atcc.org/). The cells were seeded in Dulbecco's modified Eagle's medium (DMEM) (D0819, Gibco BRL/Invitrogen) containing 10% foetal bovine serum (FBS; 10100147, Gibco BRL/Invitrogen) and 100 U/mL penicillin/streptomycin (15140122, Gibco BRL/Invitrogen), followed by culture at 37°C with 5% CO_2_.

### 2.7 Dual‐luciferase reporter assay

Possible interaction between miR‐101 and MKP‐1 was first determined in silico using a web‐based biological prediction website (https://cm.jefferson.edu/rna22/Interactive/) and the binding site was predicted. A dual‐luciferase reporter assay was then used to verify the predicted interaction between miR‐101 and MKP‐1. Artificially synthesized MKP‐1 mRNA 3′ untranslated region (UTR) fragment was inserted into pmirGLO luciferase vector (E1330, Promega) according to the predicted binding site, which was designated as MKP‐1‐wild‐type (wt). The complementary sequence mutation site of the seed sequence was designed on the basis of MKP‐1‐wt, which was inserted to the reporter plasmid, and the recombinant plasmid MKP‐1‐mutant (mut) was obtained. The correctly sequenced luciferase reporter plasmid was co‐transfected into HEK‐293T cells with miR‐101 and miR‐101‐NC, respectively. Luciferase activity was measured using a dual‐luciferase assay kit (E1910, Promega) and luminance was detected using a Promega's GLoma × 20/20 Luminometer (E5311, Shaanxi Zhongmei Biotechnology Co., Ltd.). All experiments were repeated three times independently.

### 2.8 RNA isolation and quantitation

Total RNA was extracted from the spinal cord or cells of each group after 36 hours of transfection using TRIzol reagent (10296010, Invitrogen) according to the manufacturer's protocol. The concentration, purity and integrity of the extracted RNA were determined by Nano‐Drop ND‐1000 spectrophotometry and 1% agarose gel electrophoresis. All primers were synthesized by Beijing Genomics Institute, Co., Ltd. (Table S1). The miRNA‐specific complementary DNA was synthesized using a TaqMan™ MicroRNA Reverse Transcription Kit (4366596, Applied Biosystems™) with miRNA‐specific RT primers from TaqMan MicroRNA Assay (Thermo Fisher Scientific). The expression of miR‐101 was measured by TaqMan miRNA Assay (Thermo Fisher Scientific) according to the manufacturer's instructions, and normalized to U6. The total RNA was subjected to reverse transcription quantitative polymerase chain reaction (RT‐qPCR) using a SYBR^®^ Premix Ex TaqTM II kit (RR820A, TaKaRa), with a reaction volume of 25 μL. With glyceraldehyde‐3‐phosphate dehydrogenase (GAPDH) as an internal reference, the relative expression of the gene of interest was calculated using the 2^−∆∆Ct^ method. The experiment was repeated three times independently.

### 2.9 Fluorescence in situ hybridization (FISH)

Subcellular localization and expression of miR‐101 were detected using a FISH kit (BIS‐P0001, Guangzhou Boxin Biotechnology Co., Ltd.). Rats were euthanized under anaesthesia. L4‐L6 spinal dorsal horn was fixed with 4% paraformaldehyde and made to sections and the microglial cells were allowed to grow on slides. After dehydration and drying, the sections were incubated in hybridization solution for 2 hours at room temperature. Next, the sections were incubated in hybridization solution with 8 ng/μL of FAM (488) labelled probe for miR‐101 overnight (Wuhan Servicebio Technology Co., Ltd.) at 37°C. An antagonistic miR‐101 probe was set up as a NC and all following procedures were performed according to manufacturer's instructions. Fluorescence images were obtained using a Zeiss LSM880 laser confocal scanning microscope (Leica Microsystems). The experiment was repeated three times independently.

### 2.10 Enzyme‐linked immunosorbent assay (ELISA)

The levels of inflammatory factors interleukin (IL)‐6, tumour necrosis factor‐α (TNF‐α), IL‐1β and COX‐2 in rat spinal cord and microglia samples were measured following the instructions of the Simple Step ELISA^®^ kits (ab100712, ab208348, ab100768, ab52237) purchased from Abcam. An EON spectrophotometer (BioTek Instruments) was employed to quantify the levels of the microplate at 450 nm.

### 2.11 Western blot assay

Cells were lysed on ice using 1 mL of immunoprecipitate cell lysis buffer (P0013, Beyotime) supplemented with 10 μL of phenylmethylsulphonyl fluoride (PMSF) (100 mmol/L, ST506, Beyotime) to obtain protein samples. A bicinchoninic acid (BCA) protein assay kit (P0012S, Beyotime) was adopted to determine the protein concentration of each sample. After separation by sodium dodecyl sulphate‐polyacrylamide gel electrophoresis (SDS‐PAGE), the proteins were transferred onto a membrane, which was blocked with 5% BSA at 37°C for 2 hours. Next, the membrane was incubated overnight at 4°C with primary antibodies: rabbit polyclonal antibody to GAPDH/MKP‐1/p‐p38 (ab9485/ab61201/ab4822, 1:2500/1:1000/1:1000, Abcam); rabbit monoclonal antibody to Iba‐1/CD11b/JNK/p‐JNK/ c‐Jun/pc‐Jun/ p38 (ab195261/ab133357/ab208035/ab76572/ab32137/ab32385/ab170099,1:1000/1:1000/1:2000/1:5000/1:10000/1:10000/1:5000, Abcam). Thereafter, the membrane was rinsed with Tris‐buffered saline containing 0.1% tween‐20 (TBST) and incubated with goat anti‐rabbit immunoglobulin G (IgG) (ab672, 1:20 000, Abcam) for 1 hour at room temperature. The proteins on the membrane were visualized using a photographic fixing kit (P0020, Beyotime). GAPDH was used as the internal reference and images were captured using a Bio‐Rad gel imaging system. The experiment was repeated three times independently. The grey value of the protein band of interest was determined using Image J software (NIH).

### 2.12 Immunofluorescence

Upon reaching 40%‐50% confluence, cells in each well were washed 3 times with pre‐cooled PBS (5 minutes each) and fixed with 1 mL of 95% pre‐cooled ethanol at −20°C for 30 minutes. The 95% ethanol was then removed and 1 mL of PBS containing 5% BSA was added for incubation for 60 minutes at room temperature. Then, the cells were incubated overnight at 4°C with 200 μL rabbit polyclonal antibody to CD11b (ab128797, 1 μg/mL, Abcam), rabbit monoclonal antibody to Iba‐1 (ab178847, 1:100, Abcam), rabbit monoclonal antibody to NeuN (ab177487, 1:300, Abcam), rabbit polyclonal antibody to GFAP (ab7260, 1:1000, Abcam) or rabbit polyclonal antibody to CC1 (ab15270, 1:800, Abcam) or rabbit IgG (serving as NC). Thereafter, the cells were incubated with DyLight 549‐donkey anti‐rabbit (SA5‐10064, Invitrogen) at room temperature for 2 hours without light exposure, mounted with glycerine and observed under a laser confocal microscope.

### 2.13 RNA pull‐down assay

The binding of miR‐101 to MKP‐1 mRNA was examined using the Magnetic RNA‐Protein Pull‐Down kit (20164, Pierce). After the microglial cells were routinely detached, the pellet was collected after centrifugation and RIP Lysis Buffer was added to lyse the cells, followed by incubation on ice for 30 minutes. After centrifugation at 12 000 *g* for 10 minutes at 4°C, the supernatant of the lysate was equally divided into several aliquots and stored at −80°C, and one aliquot was used as the input for the pull‐down experiment. According to the kit instructions, the biotinylated miR‐101, miR‐101‐NC, MKP‐1‐wt, MKP‐1‐mut were enriched with streptavidin‐labelled magnetic beads, which were then incubated overnight with lysis buffer at 4°C. Finally, RNA was extracted by conventional TRIzol method for purification, followed by measurement of MKP‐1 mRNA expression by RT‐qPCR.

### 2.14 Immunohistochemistry

On the 7th day post‐operation, rats were deeply anaesthetized with sodium pentobarbital (50 mg/kg, ip) and intracardially perfused with saline. Next, 4.0% paraformaldehydein 0.1 mol/L PBS (pH = 7.4, Sigma) was used for perfusion. Spinal cord segments L4‐L6 were extracted and fixed at 4°C for 12 hours in the same fixative, and then transferred to PBS containing sucrose (15%‐20%). On the next day, the segments were sliced continuously at a thickness of 30 μm. Free floating sections were subsequently stained using the standard avidin‐peroxidase complex (ABC) method. The sections were incubated overnight in primary rabbit monoclonal antibody to CD11b (ab133357, 1:250, Abcam) in 0.1 mol/L PBS containing 5% normal goat serum and re‐probed with diluted biotinylated goat anti‐rabbit IgG (1:200). The product was visualized with 0.03% hydrogen peroxide and 0.05% 3, 3′‐diaminobenzidine (DAB) solution as chromogen. The sections were then fixed on glass slides, dehydrated by gradient ethanol, dehydrated with xylene, permeabilized and mounted. The sections were observed under a brightfield Olympus BX51/BX52 microscope. Images were obtained using an Olympus DP50 digital camera and processed using the Olympus DP Image software (version 3.1).

### 2.15 Statistical analysis

Statistical analysis was performed using SPSS 21.0 statistical software (IBM Corp). The measurement data were summarized as mean ± standard deviation. Normality and homogeneity of variance were tested, and the data obeying normal distribution and homogeneity of variance between two groups were compared using unpaired *t* test. The comparison among multiple groups was performed using one‐way analysis of variance (ANOVA) or repeated measures ANOVA, followed by Tukey's post hoc test for pairwise comparison. The rank sum test was performed for data with unequal variances or skewed distribution. *P < *.01 indicated the difference was statistically significant.

## 3 RESULTS

### 3.1 MiR‐101 is highly expressed in CCI rat models

**Figure 1:**
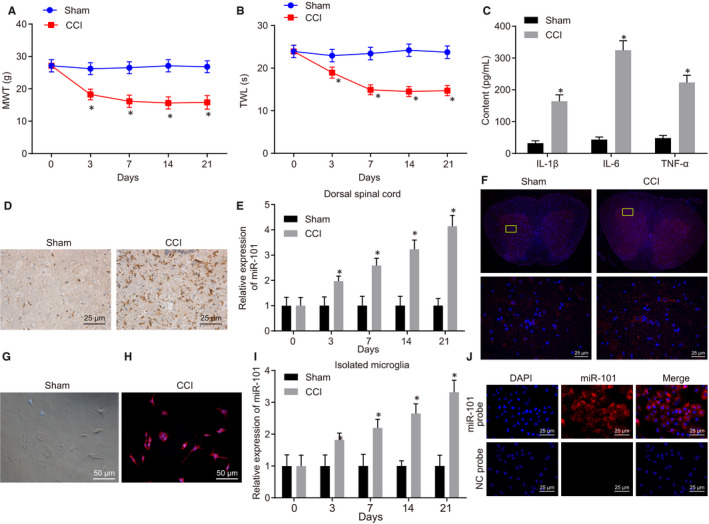
MiR‐101 is highly expressed in CCI rat models. A, MWT indicating the hypersensitivity to mechanical stimulation on the 0, 3rd, 7th, 14th and 21st days after operation in the sham‐operated rats and CCI rat models. n = 6. B, TWL indicating the hypersensitivity to thermal hyperalgesia at the 0, 3rd, 7th, 14th and 21st days after operation in the sham‐operated rats and CCI rat models. n = 6. C, levels of IL‐1β, IL‐6 and TNF‐α in the L4‐L6 segments of spinal dorsal horn in sham‐operated rats and CCI rat models on the 7th day after operation detected by ELISA. n = 3. D, immunohistochemical detection of the expression of microglial cell marker CD11b in the L4‐L6 spinal cord in the sham‐operated rats and CCI rat models on the 7th day after operation. n = 3. E, the relative expression of miR‐101 in L4‐L6 spinal cord dorsal horn of sham‐operated rats and CCI rat models at the 0, 3rd, 7th, 14th and 21st days after operation detected by RT‐qPCR. n = 3. F, miR‐101 expression in L4‐L6 spinal dorsal horn of sham‐operated rats and CCI rat models on the 7th day after operation identified using FISH assay. n = 3. G, microscopic observation from morphological characteristics of microglia isolated from the spinal cord. H, immunofluorescence assay of the isolated microglia marker CD11b expression to identify the purity of isolation. I, the expression of miR‐101 in microglial cells isolated from the L4‐L6 spinal cord of the sham‐operated rats and CCI rat models at the 0, 3rd, 7th, 14th and 21st days after operation determined by RT‐qPCR. J, the localization of miR‐101 in microglial cells isolated from the L4‐L6 spinal cord on the 7th day post‐CCI model construction detected by FISH assay. Measurement data were expressed as mean ± standard deviation. Comparison between time‐based measurements within each group was performed with repeated measures ANOVA (panels A, B, E, I), followed by Tukey's post hoc test. Comparison between two groups was performed by independent sample *t* test (panel C). * *P < *.05 vs the sham group. All cell experiments were repeated three times

Foot licking, biting or shaking after the ligation of bilateral sciatic nerves indicated the occurrence of spontaneous pain. Besides, MWT was shown to be significantly lower in the CCI group than that in the sham group, suggesting mechanical allodynia (*P < *.05) (Figure 1A). Moreover, TWL in the CCI group was significantly lower than that in the sham group, suggesting thermal hyperalgesia (*P < *.05) (Figure 1B). Notably, MWT and TWL displayed the lowest values on the 7th day after operation, suggesting that animal samples on the 7th day after CCI were sufficient for subsequent experiments. Meanwhile, the level of neuroinflammation in the spinal dorsal horn of the rats was determined by ELISA. The results showed that the levels of IL‐1β, IL‐6 and TNF‐α in the spinal dorsal horn of rats in the CCI group were significantly increased by at least 3 times versus those of rats in the sham group (*P < *.05) (Figure 1C). To determine the degree of spinal cord microglia activation, we performed immunohistochemistry experiments to detect the expression of the microglia marker CD11b in the spinal dorsal horn. The results demonstrated that on the 7th day after CCI in SD rats, CD11b expression of microglia in the spinal cord horn was significantly higher in the CCI group, suggesting that the microglial cells were much more activated in the CCI group (Figure 1D). Overall, the rats following CCI model construction developed in this study showed higher spontaneous pain, thermal pain sensitivity and mechanical allodynia, along with obvious inflammation and microglia activation in the spinal cord, indicating the successful induction of neuropathic pain in SD rats.

Further, we employed RT‐qPCR to quantify the expression of miR‐101 in L4‐L6 segments of the spinal dorsal horn at 0, 3rd, 7th, 14th, 21st days post‐CCI establishment of rats in the sham group and the CCI group. As described in Figure 1E, the expression of miR‐101 in the spinal dorsal horn of the CCI group was significantly increased on the 3rd day after operation, and gradually increased over prolonged post‐operative time (*P < *.05). In contrast, miR‐101 expression stayed the same over time course in the sham group. FISH assay also showed a significant increase in miR‐101 expression in the spinal dorsal horn on the 7th day post‐operatively in the CCI group as compared to the sham group (Figure 1F).

Under the phase contrast microscope, the majority of cells isolated from rat spinal cord were microglial cells. As shown in Figure S1, the final isolated cells contained over 90% microglial cells, about 10% cells astrocytes, few oligodendrocytes and other types of cells. The isolated microglial cells were seen as polarized amoebocytes with round‐shaped cell body which were unevenly polarized with burr‐like edges (Figure 1G,H). In addition, the microglial cells were positive for CD11b/c, indicating the successful isolation of high‐purity microglial cells from the spinal cord of experimental rats. Thereafter, RT‐qPCR was conducted to determine the expression of miR‐101 in the isolated microglial cells, the results of which presented that miR‐101 expression was significantly higher in the CCI group than that in the sham group on the 3rd day after operation (*P < *.05) (Figure 1I), and it was gradually increased with the prolonged post‐operative time, which was consistent with the results obtained from the spinal cord tissues. At the same time, we observed that miR‐101 was mainly distributed in the cytoplasm of microglial cells (Figure 1J). These results indicated that miR‐101 was highly expressed in the spinal dorsal horn and microglial cells of CCI model rats, which implied that miR‐101 was likely to promote the sensitization process of neuropathic pain.

### 3.2 MiR‐101 loss‐of‐function reduces hypersensitivity to pain in CCI rat models

**Figure 2:**
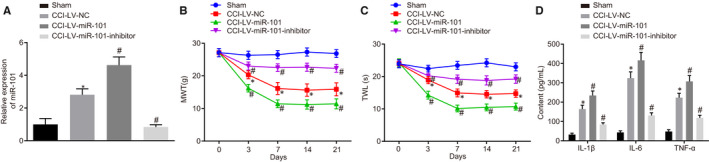
High expression of miR‐101 promotes hypersensitivity to pain in CCI rat models. A, The expression of miR‐101 in the L4‐L6 spinal cord on the 7th day after CCI induction examined by RT‐qPCR. B, MWT of rats in response to overexpressed or inhibited miR‐101 after CCI induction. C, TWL of rats in response to overexpressed or inhibited miR‐101 after CCI induction. D, Levels of inflammatory factors in the L4‐L6 spinal dorsal horn on the 7th day after CCI induction measured by ELISA. Measurement data were expressed as mean ± standard deviation. Comparison between time‐based measurements within each group was performed with repeated measures ANOVA (panels B, C), and comparison among multiple groups with one‐way ANOVA (panel A, D), followed by Tukey's post hoc test. * *P* < .05 vs the sham group. ^#^ *P < *.05 vs the CCI‐LV‐NC group. All experiments were repeated three times

As depicted in Figure 2A, lentiviral vectors overexpressing or knocking down the expression of miR‐101 were successfully constructed. Mechanical and thermal stimulation experiments showed that the degree of response was significantly higher in the CCI‐LV‐NC group than that in the sham group, indicating that CCI model was successfully developed. Compared with the CCI‐LV‐NC group, the MWT and TWL in rats in the CCI‐LV‐miR‐101 group were significantly increased, indicating a significantly higher sensitivity to the pain caused by stimulation. However, the MWT and TWL in rats in the CCI‐LV‐miR‐101‐inhibitor group were significantly decreased, indicating that these rats were less sensitive to pain (*P < *.05) (Figure 2B,C). miR‐101 overexpression was found to increase the levels of IL‐1β, IL‐6 and TNF‐α in the spinal dorsal horn of rats with CCI, and all of which were significantly decreased by miR‐101 knockdown (*P < *.05) (Figure 2D). Taken together, the results demonstrated that up‐regulation of miR‐101 promoted pain hypersensitivity in CCI rat models, whereas the down‐regulation of miR‐101 attenuated this sensitization.

### 3.3 MiR‐101 loss‐of‐function impedes lipopolysaccharide (LPS)‐induced activation of spinal cord microglia and reduces inflammation

**Figure 3:**
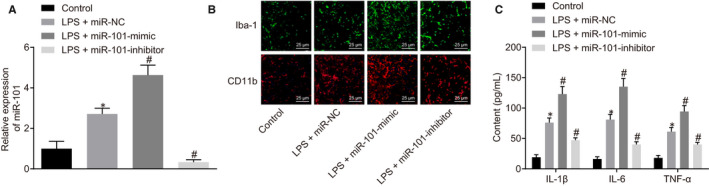
Knockdown of miR‐101 inhibits LPS‐induced activation of spinal cord microglia and reduces inflammation. A, the expression of miR‐101 in microglial cells determined by RT‐qPCR. B, the expression of Iba1 and CD11b in microglial cells detected by immunofluorescence assay. C, the levels of inflammatory factors IL‐1β, IL‐6 and TNF‐α in the spinal cord microglia determined by ELISA. Measurement data were expressed as mean ± standard deviation. Comparison among multiple groups was performed with one‐way ANOVA, followed by Tukey's post hoc test. *P < .05 vs the control group. ^#^ *P < *.05 vs the LPS + miR‐NC group. All experiments were repeated three times

As shown in Figure 3A, 0.5 ng/mL LPS induced the activation of microglia and increased the expression of miR‐101, miR‐101 was further up‐regulated or inhibited in the LPS‐treated microglial cells by miR‐101 mimic or miR‐101 inhibitor. Immunofluorescence staining clearly showed that the microglial cells of the spinal cord were activated by LPS. The LPS‐exposed cells showed a morphological change from amoebiform and long shape with asymmetrical branches to an amoeba‐like shape, with enrichment of the markers CD11b and Iba1 (Figure 3B). In the LPS‐exposed microglial cells, the secreted levels of IL‐1β, IL‐6 and TNF‐α were increased upon miR‐101 overexpression (Figure 3C). On the contrary, the secretion of those pro‐inflammatory factors IL‐1β, IL‐6 and TNF‐α in the LPS‐exposed microglial cells was inhibited by miR‐101 loss‐of‐function inhibited the secretion of these pro‐inflammatory factors, accompanied by decreased expression of CD11b and Iba1 (*P < *.05) (Figure 3B,C). These results suggested that inhibition of miR‐101 hindered LPS‐induced activation of spinal cord microglia and reduced inflammation.

### 3.4 MiR‐101 binds to the 3′UTR of MKP‐1 mRNA

**Figure 4:**
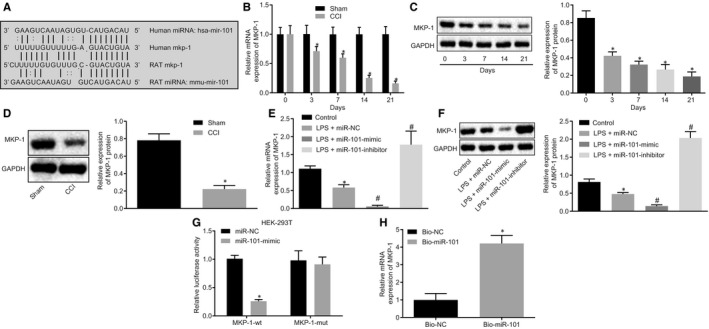
MKP‐1 is a target gene of miR‐101. A, Predicted binding sites between miR‐101 and MKP‐1 using the miRanda and TargetScan databases. B, mRNA expression of MKP‐1 in the L4‐L6 spinal dorsal horn of sham‐operated rats and CCI rats determined using RT‐qPCR. C, Western blot analysis of MKP‐1 protein in L4‐L6 spinal dorsal horn from CCI rats. D, Western blot analysis of MKP‐1 protein in microglial cells isolated from L4‐L6 spinal cord on the 7th day after operation in sham‐operated rats and CCI rat models. E, the expression of MKP‐1 mRNA in isolated microglial cells determined using RT‐qPCR. F, Western blot analysis of MKP‐1 protein in LPS‐exposed microglial cells. G, The binding of miR‐101 to MKP‐1 verified using dual‐luciferase reporter assay. H, The binding of miR‐101 to MKP‐1 verified using RNA pull‐down assay. Measurement data were expressed as mean ± standard deviation. Comparison between time‐based measurements within each group was performed with repeated measures ANOVA (panel B), and comparison among multiple groups with one‐way ANOVA (panel E), followed by Tukey's post hoc test. Comparison between two groups was performed by independent sample *t* test (panel G, H). *P < .05 vs the sham or control group. ^#^ *P < *.05 vs the LPS + miR‐NC group. All experiments were repeated three times

The miRNA target prediction websites (miRanda and TargetScan) predicted the complementary sequence between miR‐101 and MKP‐1 3′UTR both in the human genome and the rat genome (Figure 4A). As miR‐101 was highly expressed in CCI model rats (Figure 1E,F), we thus hypothesized that MKP‐1, a putative target gene of miR‐101, might be down‐regulated in CCI rat models. The mRNA and protein expression of MKP‐1 was significantly decreased in the spinal dorsal horn of rats in the CCI group on the 3rd day post‐operation, and the expression was decreased gradually with the prolonged post‐operative time (*P < *.05) (Figure 4B,C). In the isolated microglia from the L4‐L6 spinal cord on the 7th day post‐operation, MKP‐1 protein expression was also observed to be significantly reduced (Figure 4D). Similarly, the mRNA and protein expression of MKP‐1 in LPS‐activated microglial cells was significantly reduced. Overexpression of miR‐101 further decreased the mRNA and protein expression of MKP‐1 in activated microglial cells. In contrast, upon knockdown of miR‐101 expression in the activated microglial cells, the mRNA and protein expression of MKP‐1 was significantly elevated, indicating that miR‐101 negatively regulated the expression of MKP‐1 (*P < *.05) (Figure 4E,F). Furthermore, dual‐luciferase reporter and RNA pull‐down assays verified that miR‐101 specifically bound to the 3'UTR of MKP‐1 (*P < *.05) (Figure 4G,H). Taken together, these data indicated miR‐101 could down‐regulate MKP‐1 expression in microglial cells by binding to the 3′UTR of MKP‐1.

### 3.5 MiR‐101 enhances inflammation in spinal cord microglia by inhibiting MKP‐1 expression

**Figure 5:**
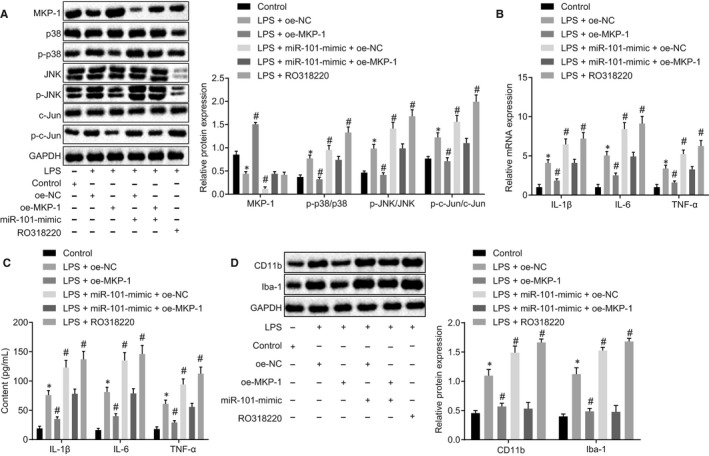
MiR‐101 enhances inflammation in spinal cord microglia by inhibiting MKP‐1 expression in the MAPK signalling pathway. A, Western blot analysis of the phosphorylated p38/JNK/c‐Jun proteins in the spinal cord microglial cells. B, mRNA expression levels of inflammatory factors in the spinal cord microglial cells determined by RT‐qPCR. C, Levels of inflammatory factors in spinal cord microglial cells detected by ELISA. D, Western blot analysis of CD11b and Iba‐1 proteins in the spinal cord microglia. Measurement data were expressed as mean ± standard deviation. Comparison among multiple groups was performed with one‐way ANOVA, followed by Tukey's post hoc test. *P < .05 vs the control group. ^#^ *P < *.05 vs the LPS + oe‐NC group. All experiments were repeated three times

Next, we explored whether miR‐101 can affect the MAPK signalling pathway by inhibiting MKP‐1. Western blot analysis (Figure 5A) showed that the extent of p38, JNK and c‐Jun phosphorylation in LPS‐activated microglial cells was significantly higher than that in control cells without LPS stimulation, indicating that the p38 MAPK/JNK signalling pathway was activated (Figure 5B). This activation led to increased secretion of inflammatory factors IL‐1β, IL‐6, TNF‐α and COX‐2 in microglial cells (Figure 5C). In the LPS‐activated microglial cells, the overexpression of MKP‐1 reduced the extent of p38, JNK and c‐Jun phosphorylation and inhibited cellular inflammation. In the microglial cells treated with miR‐101‐mimic and oe‐NC or those treated with the MKP‐1 inhibitor RO318220, the expression of MKP‐1 was inhibited, and the MAPK signalling pathway was activated, coupled with aggravated cellular inflammation. Notably, the restoration of MKP‐1 in LPS‐induced microglial cells blocked the MAPK signalling pathway and attenuated inflammation that was aggravated by miR‐101 (*P < *.05). MKP‐1 overexpression thus counteracted the regulatory effects of miR‐101 (Figure 5B,C). This effect was also reflected in the expression of the microglia activation markers CD11b and Iba1 (Figure 5D). Together, these findings indicated that miR‐101 inhibited MKP‐1 to activate the MAPK signalling pathway in spinal cord microglial cells, thereby increasing inflammation.

### 3.6 MiR‐101 aggravates pathological pain in CCI rat models through suppression of MKP‐1 in the MAPK signalling pathway

**Figure 6:**
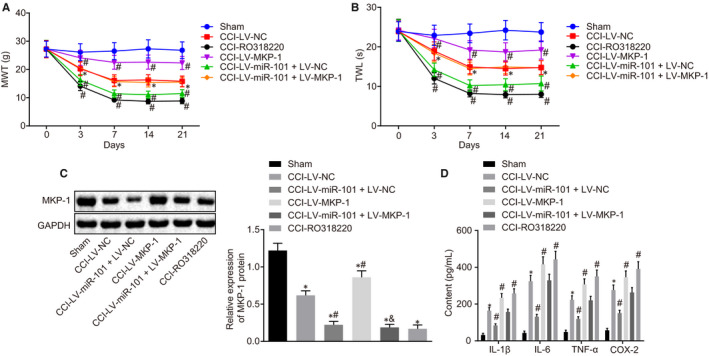
MiR‐101 aggravates neuropathic pain by inhibiting MKP‐1 in the MAPK signalling pathway in CCI rat models. A, MWT of rats following CCI model construction. B, TWL of rats following CCI model construction. C, Western blot analysis of MKP‐1 protein in the L4‐L6 spinal cord on the 7th day after CCI model construction. D, Levels of inflammatory factors in the L4‐L6 spinal cord dorsal horn of rats on the 7th day after CCI model construction detected by ELISA. Measurement data were expressed as mean ± standard deviation. Comparison between time‐based measurements within each group was performed with repeated measures ANOVA (panels A, B), and comparison among multiple groups with one‐way ANOVA (panel D), followed by Tukey's post hoc test. *P < .05 vs the sham group. ^#^ *P < *.05 vs the CCI‐LV‐NC group. All experiments were repeated three times

In CCI rat models, the overexpression of MKP‐1 diminished the allodynia response reflected by significant increases in MWT and TWL (Figure 6A,B). CCI rats in the LV‐miR‐101 + LV‐NC group and the CCI rats injected with the MKP‐1 inhibitor RO318220 displayed lower MWT and TWL than those in the LV‐NC group, indicating higher sensitivity to pain stimuli in the presence of miR‐101 or absence of MKP‐1. More importantly, the CCI rats in the LV‐miR‐101 + LV‐MKP‐1 group showed weakened sensitivity to stimuli as compared with those in the LV‐miR‐101 + LV‐NC group (*P < *.05) (Figure 6A,B). The results of Western blot analysis and ELISA assay illustrated that as compared with the sham group, the expression of MKP‐1 was decreased in the spinal dorsal horn of rats in the CCI group, and correspondingly, the levels of inflammatory factors were significantly increased. Furthermore, the expression of MKP‐1 was up‐regulated in the spinal dorsal horn of CCI rats in the LV‐MKP‐1 group in comparison to the LV‐NC group, accompanied by decreased inflammation (*P < *.05). On the contrary, the LV‐miR‐101 + LV‐NC group displayed reduced expression of MKP‐1 and enhanced inflammation in the spinal dorsal horn of CCI rats when compared with the LV‐NC group. Inflammation was inhibited and MKP‐1 expression was rescued in the LV‐miR‐101 + LV‐MKP‐1 group vs the LV‐miR‐101 + LV‐NC group (*P < *.05) (Figure 6C,D). Taken together, the results demonstrated that miR‐101 could promote pain hypersensitivity in CCI rat models and aggravate neuropathic pain by inhibiting MKP‐1 in the MAPK signalling pathway.

## 4 DISCUSSION

Chronic neuropathic pain caused by spinal cord injury commonly inflicts a persistent and considerable adverse impact on patients’ quality of life, which makes effective pain management an urgent and significant necessity.^21^ Existing evidence suggests that miR‐101 controls various developmental processes in adult neural networks by regulating a highly interconnected gene network involved in the initial development of neural nets, suggesting new insights into brain development and potential neurological treatment strategies.^22^ Therefore, we performed this study to examine the promotive effect of miR‐101 in pain hypersensitivity and inflammation in a rat model of CCI. The key findings indicated that miR‐101 was responsible for pain hypersensitivity in CCI through down‐regulating MKP‐1 expression in the MAPK signalling pathway in microglial cells.

After the operation of sciatic nerve ligation, CCI rat models presented with significant spontaneous pain, thermal hyperalgesia and mechanical allodynia, accompanied by elevated levels of IL‐1β, IL‐6 and TNF‐α in the spinal dorsal horn. The robust secretion of pro‐inflammatory cytokines and the activation of spinal cord microglial cells have been documented to stimulate the persistence and progression of neuropathic pain following peripheral nerve damage.^3^, ^23^ A previous study demonstrated that the exacerbated inflammatory response is linked with activated microglial cells following traumatic brain injury.^24^ In neuropathic pain, microglia plays an important role in the process of pain sensitization and thus comprises a promising target for neuropathic and post‐operative pain, and morphine tolerance.^25^ The secreted inflammatory mediators act to stimulate induction and cascade expansion during pain transmission between microglial cells and neurons.^26^

Our results highlighted that miR‐101 was highly expressed in the spinal cord dorsal horn in CCI rat models and the microglial cells isolated from these rats. These results are consistent with recent evidence indicating that miRNAs have critical roles in the induction and progression of chronic pain.^9^ More specifically, evidence has demonstrated that miRNAs are involved in the pathophysiologic processes of spinal cord injury and can be considered potential therapeutic targets.^27^ In addition, reports indicate that miR‐101 expression is clearly associated with inflammation and it mediates the secretion of inflammatory factors.^28^, ^29^ Our study also demonstrated that overexpression of miR‐101 enhanced inflammation in LPS‐exposed spinal cord microglial cells and in CCI rat models, as reflected by the increased expression of IL‐1β, IL‐6, TNF‐α and COX‐2. In addition, the findings from the present study demonstrated that miR‐101 promoted hypersensitivity to pain in CCI rats with reduced MWT and TWL. MiR‐101a is known to modulate microglial cell morphology and function and also to enhance the production of IL‐6 and TNF‐α from microglial cells in response to LPS treatment.^12^ A possible role of miR‐101 in neuropathological disorders has also been documented in hippocampal neurons, where it can expedite the production of pro‐inflammatory cytokine IL‐1β by modulation of the amyloid precursor protein.^30^ On the other hand, the up‐regulation of IL‐6 and TNF‐α is shown to promote the expression of miR‐101, which subsequently augments intracellular cholesterol retention under inflammation conditions.^31^ This highlights another possible mechanism associated with the neuroinflammation, which warrants further verification.

Furthermore, we verified MKP‐1 to be a target gene of miR‐101. It is well established that MKP‐1 is a member of the dual‐specificity phosphatase family that deactivates MAPKs.^32^ Our results showed that miR‐101 targets the 3'UTR of MKP‐1 and inhibits its expression. miR‐101 similarly modulates immune responses of macrophages to LPS by targeting MKP‐1.^33^ Furthermore, it was suggested that miR‐101 can bind to MKP‐1 mRNA 3′UTR and the MAPK/MKP‐1/miR‐101 axis thus plays a regulatory role in the related immune response and pathogenesis of systemic lupus erythematosus.^34^ However, whether or how miR‐101 aggravates neuropathic pain in CCI rat models and enhances inflammation in spinal cord microglia has not been investigated. Our data provide direct evidence that miR‐101 might promote pain hypersensitivity in rats with CCI *via* MKP‐1 suppression and activation of the MAPK signalling pathway. In agreement with our findings, LPS‐induced inflammatory injury is reportedly attenuated by down‐regulated miR‐101 expression and inactivated JNK signalling pathway *via* the up‐regulation of MKP‐1 expression.^34^ Finally, the anti‐inflammatory effect on LPS‐activated macrophages could be achieved primarily through the mediation of the miR‐101/MKP‐1/MAPK axis, with miR‐101 as a negative regulator of MKP‐1 expression.^15^ Additional work is needed to confirm whether miR‐101 regulates MKP‐1 expression through mRNA degradation or by translational regulation.

**Figure 7:**
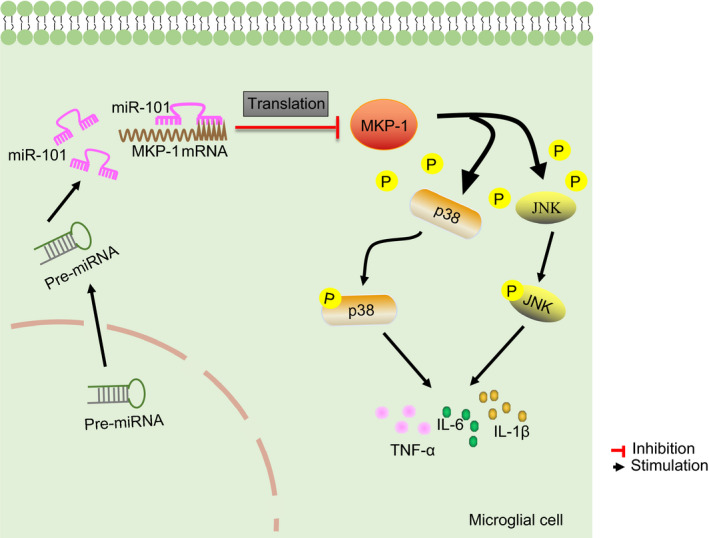
A schematic map depicting the role of miR‐101 in pain hypersensitivity in rats with CCI. The overexpression of miR‐101 in rat spinal cord microglial cells promotes an inflammatory response in microglial cells by inhibiting the expression of MKP‐1, a key factor in the MAPK signalling pathway, thus aggravating neuropathic pain in CCI rat models

Based on the aforementioned evidence, the pain hypersensitivity and inflammation in rats with CCI could be accelerated by miR‐101‐mediated MKP‐1 inhibition in microglial cells (Figure 7). miR‐101 and MKP‐1 may therefore have the potential to serve as therapeutic targets for chronic neuropathic pain management. More generally, the miRNA‐based clinical trials may provide potential therapeutic tools for chronic neuropathic pain, which enable improved categorization of risk, prognosis and possibly translate to personalized therapy. However, future researches should confirm the findings with larger patient cohorts and identify the best strategy to limit the side effects.

## CONFLICTS OF INTERESTS

None.

## AUTHOR CONTRIBUTION

**Shuang Qiu:** Conceptualization (equal); Data curation (equal); Formal analysis (equal); Methodology (equal); Resources (equal). **Benjuan Liu:** Conceptualization (equal); Data curation (equal); Formal analysis (equal); Methodology (equal); Resources (equal); Software (equal). **Yanshuai Mo:** Conceptualization (equal); Writing‐original draft (equal); Writing‐review & editing (equal). **Xueqin Wang:** Conceptualization (equal); Methodology (equal); Writing‐original draft (equal); Writing‐review & editing (equal). **Lina Zhong:** Conceptualization (equal); Writing‐original draft (equal); Writing‐review & editing (equal). **Xiao Han:** Conceptualization (equal); Writing‐review & editing (equal). **Fuli Mi:** Conceptualization (equal); Writing‐review & editing (equal).

## ETHICAL APPROVAL

Animal experiments were performed with the approval of the Ethics Committee of Linyi People's Hospital and in accordance with *the Guide for the Care and Use of Laboratory animals* published by the US National Institutes of Health. All efforts were made to minimize the suffering of the animals included in the study.

## CONSENT FOR PUBLICATION

Consent for publication was obtained from the participants.

## Supporting information

Fig S1

Table S1

## Data Availability

The datasets generated/analysed during the current study are available.
