# Supplementary material for: MiR‐101 promotes pain hypersensitivity in rats with chronic constriction injury via the MKP‐1 mediated MAPK pathway
Source: J Cell Mol Med. 2020 Jul 13;24(16):8986–97. doi: 10.1111/jcmm.15532 (PMC7417728; doi:10.1111/jcmm.15532)
Supplement: Supplementary file 2 — Table S1 [file JCMM-24-8986-s002.docx]

**Supplemental Table 1.** Primer sequences for RT-qPCR.

| Gene | Primer sequence (5'-3') |
| --- | --- |
| miR-101 | Forward: UACAGUACUGUGAUAACUGAA |
|  | Reverse: CAGUUAUCACAGUACUGAAUU |
| U6 | Forward: GCTACCTCTCAATCCCACCG |
|  | Reverse: CAATCAGCGAGCAGCTCAAC |
| MKP-1 | Forward: GAGCTGTGCAGCAAACAGTC |
|  | Reverse: CTTCCGAGAAGCGTGATAGG |
| GAPDH | Forward: TGCACCACCAACTGCTTAG |
|  | Reverse: GGATGCAGGGATGATGTTC |
